# Insights into selective hydrogenation of levulinic acid using copper on manganese oxide octahedral molecular sieves

**DOI:** 10.1098/rsos.220078

**Published:** 2022-07-27

**Authors:** Nayan J. Mazumdar, Gunjan Deshmukh, Anna Rovea, Praveen Kumar, Miryam Arredondo-Arechavala, Haresh Manyar

**Affiliations:** ^1^ School of Chemistry and Chemical Engineering, Queen's University Belfast, David-Keir Building, Stranmillis Road, Belfast BT9 5AG, UK; ^2^ School of Maths and Physics, Queen's University Belfast, David-Keir Building, Stranmillis Road, Belfast BT9 5AG, UK

**Keywords:** hydrogenation, levulinic acid, OMS-2, γ-valerolactone, copper nanoparticles

## Abstract

Selective hydrogenation of levulinic acid (LA) to γ-valerolactone (GVL) was studied using copper on manganese oxide octahedral molecular sieve (OMS-2) as catalysts. A range of copper supported on OMS-2 catalysts was prepared using the modified wet-impregnation technique and characterized thoroughly using powder X-ray diffraction, inductively coupled plasma optical emission spectroscopy metal analysis, Fourier transform infrared, high-resolution transmission electron microscopy and N_2_ sorption analyses. Process parameters for selective hydrogenation of LA to GVL were optimized using the design of experiment (DoE) approach with response surface methodology comprising a central composite design. Using the optimized conditions (190°C reaction temperature, 20 bar H_2_ pressure and 20 wt% Cu loading on OMS-2), up to 98% yield of GVL could be achieved in water as a solvent. Based on DoE, H_2_ pressure had the most influence on GVL selectivity followed by catalyst loading used for the hydrogenation of LA. The response surface methodology model also showed synergistic effect of reaction temperature and H_2_ pressure on the yield of GVL. 20 wt% Cu/OMS-2 catalysts were re-used up to four cycles and showed noticeable loss of activity after the first cycle due to observed leaching of loose Cu species, thereafter the activity loss diminished during subsequent recycles.

## 1. Introduction

Global energy consumption in 2019 was 65 Petawatt hours (PWh) [1]. With the growth of energy demand across the world, the energy-related carbon dioxide emissions have increased to 36.4 billion tons [2]. Currently, more than 100 countries are pursuing the 2050 Net Zero targets [3]. Arriving at net-zero will require broad changes across the energy sector for transforming to the cleaner alternatives such as biomass derived fuels, wind, solar and tidal energy. Presently, with the declining crude-oil reserves and increasing need for petroleum, it is crucial to develop energy-efficient methods to produce fuels and value-added chemicals. One of the biggest challenges of the twenty-first century is the efficient transition from fossil fuels to alternative resources of energy. Biomass, which is regarded as a vast feedstock and a sustainable power source, has drawn in much consideration for the manufacture of fine synthetic substances and platform chemicals. Cellulose, hemicellulose, and lignin, which are the structural units of biomass, can be converted to a wide range of platform molecules and value-added chemicals via chemical, enzymatic hydrolysis [4]. Lignin and hemicellulose are usually converted to produce xylitol, furfural, levulinic acid (LA) and other fuel additives.

**Scheme 1.**
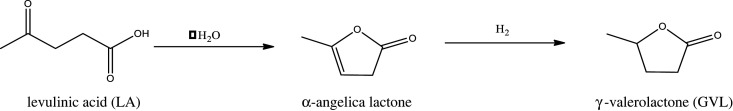
Reaction pathway for hydrogenation of LA to GVL.

LA has been listed as one of the top-12 significant building blocks by the United States Department of Energy and is designed as an intermediate platform chemical for synthesis of different value-added chemicals having a wide range of applications as fuel additives, solvents, plasticizers and pharmaceuticals [5]. LA is a C_5_ compound containing ketone and carboxylic acid as two functional groups, which are used for the production of a wide range of bio-chemicals such as γ-valerolactone (GVL), 2-methyltetrahydrofuran, 1,4-pentadiol, angelica lactone and others. GVL has a wide range of applications such as fuel additives, green solvents, polymers and as a feedstock to produce other chemicals such as pentanoic acid, valeric esters and C_8_ alkanes which can be used as jet fuel. GVL shows excellent physico-chemical properties such as low toxicity, biodegradability, high energy density and excellent fuel properties for direct use as a gasoline additive. Moreover, GVL can be used as a precursor to produce fuel range hydrocarbons. In recent years, GVL production has attracted great interest in the biorefinery industry, where the lignocellulosic-derived biomass is first converted to furfural, which can subsequently be transformed to LA via furfuryl alcohol. The process for furfural to LA conversion is well established, for instance, there are a few demonstration plants already in operation using Biofine and Dibanet technologies, but the process for LA to GVL conversion still needs to be thoroughly investigated for potential scale-up. In current practice, the LA to GVL process has only been studied at the bench scale. The transformation of LA to GVL occurs via two consecutive steps of dehydration of LA to angelica lactone (α-AL) followed by hydrogenation to GVL (scheme 1).

In recent years, a number of catalytic transformations using different catalysts, solvents and hydrogen donors have been investigated for the conversion of LA to GVL, among which the catalysts based on noble metals like ruthenium (Ru), palladium (Pd) and platinum (Pt) are the most widely explored group, mainly due to their attractive property of high activity towards carbonyl hydrogenation [6–9]. But due to the high cost and scarcity of these precious metals, there is a still need for development of earth abundant metal-based catalysts. GVL being an excellent solvent is moderately priced at US$ 2–15/kg [10]. Hence, the transition-metal-based catalysts are much more attractive for their low-cost, easy availability and better environmental sustainability. Copper is one of the most easily available abundant metals and is a prospective substitute for rare-earth and precious noble metals. Recently, many copper-based catalysts with various supports have been used for the transformation of LA to GVL via vapour and liquid-phase hydrogenation [11]. However, most Cu-based catalysts require high reaction temperatures and hydrogen pressures and show low to moderate yield of GVL. For instance, Upare *et al.* demonstrated the use of Cu/SiO_2_ catalyst for LA conversion to GVL with vapour phase reaction at 265°C using 1,4-dioxane as a solvent. They observed the formation of 1.4-pentanediol with increase in H_2_ pressure resulting in decreased selectivity to GVL [12]. Putrakumar and co-workers have reported the use of copper on γ-Al_2_O_3_ catalysts in a fixed bed glass reactor to achieve a moderate yield of GVL (40–80%). Furthermore, it was reported that the selectivity of GVL was directly related to Cu dispersion on the support [7]. Obregón *et al.* reported 96% GVL yield at 250°C, at 65 bar H_2_ pressure using commercial Al_2_O_3_ supported monometallic and bi-metallic (Ni, Cu) catalysts for the LA hydrogenation reaction. The coexistence of two metals, and Ni Cu, resulted in high conversion and minimum by-product formation [13]. Similarly, Hengne *et al.* achieved high selectivity of GVL using Cu/Al_2_O_3_ as a catalyst; however, the process requires high temperature (200°C) and high H_2_ pressure (35 bar). Also, they observed significant copper leaching in water attributed to the formation of copper-carboxylate [14]. Ishikawa *et al.* reported copper nanoparticles supported on ZrO_2_ for the hydrogenation of LA at 200°C, 35 bar H_2_ with high GVL yield, and observed steady decline in catalytic activity over a reusability study [15]. Similarly, Jones *et al*. studied a series of Cu-ZrO_2_ catalysts at 200°C and 35 bar H_2_ pressure with completion of reaction in 3 h. However, they also observed a decrease in catalytic activity after subsequent recycles. Copper leaching was minimal (from MPAES analysis) and the possible reason for the catalyst deactivation was assumed to be the sintering of Cu particles under harsh operating conditions [16]. The activity of Cu-supported catalysts could be further investigated at mild reaction conditions using water as a solvent. In this context, the use of reducible metal oxide support such as manganese oxides containing lattice oxygen vacancies could play a key role in the design of efficient and robust catalysts. Previously, we have shown that manganese oxide octahedral molecular sieves (OMS-2) materials are highly efficient and selective catalysts for liquid-phase hydrogenation of α,β-unsaturated aldehydes and ketones [17]. Using DFT calculations, we showed that the hydrogen dissociation on OMS-2 was a water-assisted process. Recently, in our group, we have demonstrated the versatile catalytic applications of manganese oxide OMS-2-based catalysts for liquid-phase hydrogenations as well as oxidation reactions [18–22]. OMS-2 shows exceptional structural attributes like porous structure, different valency of Mn ions (3^+^, 4^+^) and high lattice oxygen vacancies. To the best of our knowledge, the combination of Cu using manganese oxide as a support has not yet been studied in this reaction, additionally, using water as a solvent overall makes this a green and sustainable catalytic process. In the present work, we demonstrate that metal-doped OMS-2 catalysts can be used in selective liquid-phase hydrogenation of LA to yield GVL with high activity.

## 2. Material and methods

### 2.1. Chemicals

All chemicals were of AR grade and used without any further purification. LA (98%), copper nitrate hemi-pentahydrate [Cu(NO_3_)_2_ • 2.5H_2_O] (98%) were procured from Alfa Aesar. GVL (99%), tetrahydrofuran (THF) (99.9%), α-angelica lactone (α-ΑL) (98%), potassium permanganate and maleic acid (98%) were purchased from Sigma Aldrich.

### 2.2. Preparation of catalysts

#### 2.2.1. Preparation of octahedral manganese oxide octahedral molecular sieve molecular sieves

OMS-2 was synthesized using the modified sol–gel method reported previously [17]. In a typical preparation, the required amount of KMnO_4_ was added in deionized water and continuously stirred for 60 min at room temperature to completely dissolve KMnO_4_. To this solution, maleic acid (3 : 1 mole ratio) was slowly added and the mixture was stirred for another 3 h. A dark brown-coloured gel formed, which released water due to syneresis and it had approximately 50% H_2_O by volume of water on top of it. The resultant gel was allowed to settle, and the top layer was decanted off. Deionized water was added, and the mixture was stirred again for 10 min. The resultant solution was washed 3–4 times until it was neutral. The mixture was vacuum filtered to remove the excess water and dried overnight in an oven at 120°C. The resultant OMS-2 material was crushed into a fine powder and a 45-micron mesh sieve was used to remove any lumps. The as-synthesized OMS-2 was then calcined in air for 6 h at 450°C.

#### 2.2.2. Synthesis of Cu nanoparticles supported on octahedral molecular sieve

Cu nanoparticles with varying wt% supported on OMS-2 were prepared using a modified wet-impregnation method. In a typical synthesis, the required amount of Cu(NO_3_)_2_ • 2.5H_2_O precursor salt was dissolved in deionized water. The solution was stirred for 30 min to obtain a clear blue solution, the required amount of OMS-2 was added, and this mixture was stirred for another 3 h using a magnetic stirrer at 400–450 r.p.m. Subsequently, the temperature was set to 70°C to slowly evaporate the water. The obtained slurry was then dried overnight in the oven at 110°C. The as synthesized Cu/OMS-2 catalysts were crushed to fine powder using a mortar and pestle and subsequently calcined at 450°C for 6 h in air using a tubular furnace.

### 2.3. Catalyst characterization

All the prepared catalysts, copper (5–20 wt%) supported on OMS-2, were characterized using powder X-ray diffraction (XRD), Fourier transform infrared (FTIR) spectroscopy, Brunauer–Emmett–Teller (BET) surface area analysis, inductively coupled plasma optical emission spectroscopy (ICP-OES) and high-resolution transmission electron microscopy. XRD measurements were made with CuK *α-*radiation (1.5405 Å) using a PANalytical X'PERT PRO MPD diffractometer equipped with reflection geometry, a NaI scintillation counter, a curved graphite crystal monochromator and a nickel filter. The scattered intensities were collected from 5 to 80° (2*θ*) by scanning at 0.0178° (2*θ*) steps with a counting time of 0.5 s. FTIR spectra of samples were analysed on a Perkin–Elmer FTIR spectrometer with 32 scans per sample. The surface area, total pore volume and average pore diameter were measured by N_2_ adsorption-desorption isotherms at 77 K using Micromeritics ASAP 2020. The pore size was calculated on the adsorption branch of the isotherms using the Barrett–Joyner–Helenda method, and the surface area was calculated using the BET method. The morphology, crystallinity and chemical analysis of Cu-doped catalysts were performed on a transmission electron microscope (TEM). Thermo Fisher Talos F200X G2 in TEM and scanning TEM (STEM) mode operated at 200 kV, equipped with an FEG (field emission gun) and four in-column Super-X energy-dispersive X-ray spectrometer detectors having a total collection angle of approximately 0.9 sr. Metal analysis was performed using a Perkin–Elmer Optima 4300 ICP-OES to measure the bulk Cu content in the catalysts, as well as to check for the leaching of Cu in the reaction filtrate. The H_2_ temperature-programmed reduction (TPR) analysis was performed using the Micromeritics AutoChem II Instrument.

### 2.4. Catalytic activity

LA hydrogenation experiments were carried out in a 100 ml Autoclave Engineers' high-pressure reactor, which had a pressure range of up to 200 bar and a maximum temperature limit of 200°C. In a typical experiment, the reactor was charged with 100 mg of catalyst, 20 ml distilled water and purged four times with H_2_ to remove air, and then agitated at 1500 r.p.m. For catalyst pre-reduction, the reactor was heated to 160°C, resulting in approximately 5 bar autogenous pressure and further pressurized with additional 10 bar H_2_ pressure for 1 h. After pre-reduction, 1 g LA and 10 ml of distilled water were charged, and the reactor was purged again four times with H_2_. The autoclave reactor was then heated to 160°C, pressurized with additional 10 bar H_2_ pressure, and first reaction sample, which corresponds to *t* = 0 was collected. Thereafter, small aliquots were collected from the reaction mixture at regular intervals. The samples collected for analysis were filtered and analysed using Perkin–Elmer Clarus 500 GC equipped with a Zebron ZB-Wax column (30 m, 0.32 mm and 0.25 mm) and an FID detector. THF was used as an internal standard for GC calibrations. For catalyst reusability studies, after completion of each cycle, the reaction mixture was removed by decantation, and the recovered catalyst was re-used in the subsequent recycles without any further regeneration. Fresh quantities of LA and water were added to the reactor for each recycle.

## 3. Results and discussions

### 3.1. Catalyst characterization

Catalyst characterization studies were performed in order to identify the morphological and structural properties of the catalysts which were synthesized for the hydrogenation of LA to GVL. Table 1 summarizes the results obtained from BET surface area and pore volume of the Cu-doped catalysts along with the CuO particle size calculated by the Scherrer's equation and TEM analysis. Table 1 also compares the theoretical and measured Cu metal doping on OMS-2 from ICP-OES analysis.

**Table 1.** Comparison of ICP, BET and STEM data.

catalyst	Cu loading (wt%)		BET surface area (m^2^/g)	BET pore volume (cm^3^/g)	average CuO crystallite size (nm)^a^	average CuO nanoparticle size (nm)^b^
	theoretical	measured using ICP				
OMS-2	—	—	76.1	0.25	—	—
5% Cu/OMS-2	5	5.06	27.3	0.15	27	27
7.5% Cu/OMS-2	7.5	7.45	28.2	0.10	27	26
15% Cu/OMS-2	15	15.76	17.6	0.07	24	25
20% Cu/OMS-2	20	20.91	20.7	0.11	18	27

^a^Measured using Scherrer equation from PXRD patterns.

^b^Measured using HR-TEM.

The OMS-2 support has a BET surface area of 76 m^2^ g^−1^, which decreased to 27.3 m^2^ g^−1^ after incorporation of 5 wt.% Cu on the surface, and further to 20.7 m^2^ g^−1^ after incorporation of 20 wt.% Cu on the surface. In addition, the pore volume of OMS-2 decreased after impregnation of Cu on the surface, which is reasonable on account of surface coverage by CuO nanoparticles. The metal percentage loading measured using ICP-OES analysis confirmed the impregnation of expected Cu loadings on the surface of OMS-2. The crystallite size of CuO was calculated from the XRD peak observed at 2*θ* = 38.9° using Scherrer's equation and compared with similar values observed from TEM analysis (table 1).

**Figure 1.**
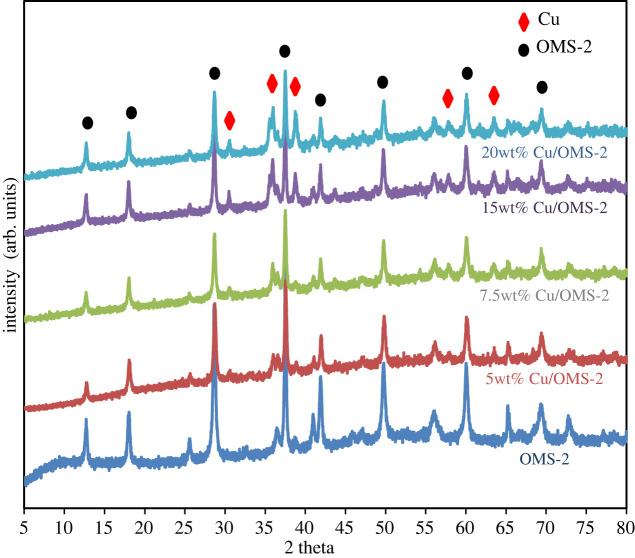
X-ray diffraction patterns of various Cu/OMS-2 catalyst samples.

**Figure 2.**
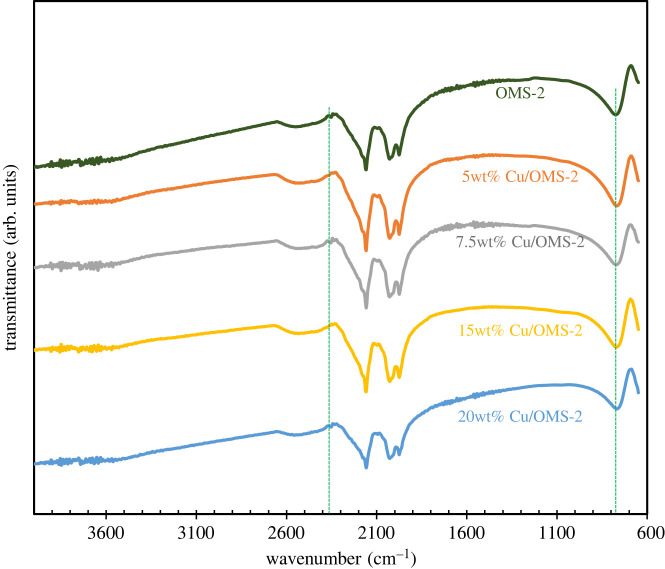
FTIR spectra of OMS-2 and Cu/OMS-2 catalysts prepared by wet-impregnation method.

The XRD patterns of OMS-2 and different Cu/OMS-2 catalysts with varying Cu loadings (wt%) are shown in figure 1. The diffraction peaks at 2*θ* = 12.6°, 17.9°, 28.7°, 38.9°, 41.9°, 49.9° and 60.1° correspond to the crystalline phase of KMn_8_O_16_ which confirms that the synthesized OMS-2 has a cryptomelane-type structure [23]. The broader diffraction peaks imply smaller particle size with large surface areas of the catalyst. The intensity of CuO diffraction peaks increased proportionately with increasing Cu loading from 5 to 20 wt%, while the peaks corresponding to OMS-2 remained the same. The FTIR spectra of OMS-2 and various wt% of Cu doped on OMS-2 catalysts are shown in figure 2. The peaks observed at 1631, 719 and 714 cm^−1^ can be attributed to the Mn-O vibrations of the MnO_6_ octahedral framework of OMS-2 [24], while the peak at 2373 cm^−1^ can be assigned to stretching vibrations of Mn_2_O_3_ [25].

**Figure 3.**
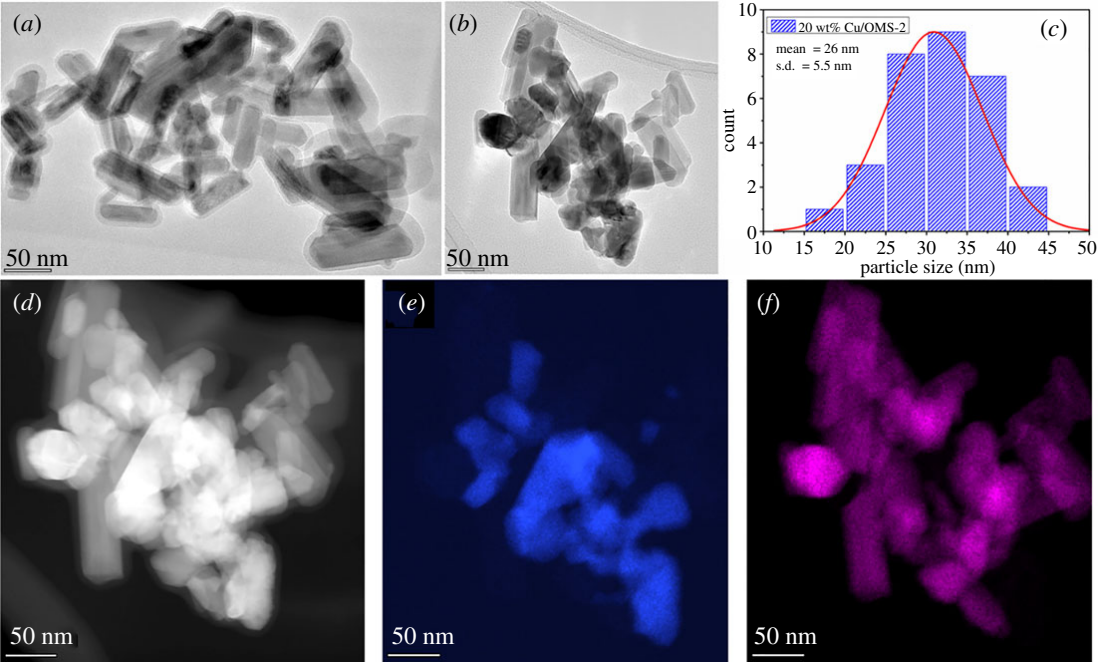
20 wt% Cu/OMS-2 (*a*) and (*b*) representative bright-field TEM images, (*c*) histogram showing the CuO particle size distributions, (*d*) representative HAADF-STEM image, EDX elemental maps for (*e*) Cu and (*f*) Mn, respectively.

The catalyst 20 wt% Cu/OMS-2 was further analysed by TEM techniques (figure 3). Overall, a large size distribution was found for this sample (figure 3*a*–*c*). The average size of CuO particles was measured to be 26 nm with an s.d. of 5.5 nm; this was obtained by measuring greater than 30 individual particles (avoiding big agglomerations). These particles exhibit predominantly spherical and elongated shapes. On the other hand, MnO_2_ particles were seen to be rod-shaped, with a large distribution in rod lengths (40–300 nm), with diameters of 20–40 nm, having aspect ratios between 2 and 14. The phase of MnO_2_ was identified as α-MnO_2_ and CuO was identified as monoclinic, based on high-resolution TEM images and FFT analysis (not shown here). STEM-EDX further confirmed that CuO crystals are evenly distributed over the MnO_2_ nanorods, where higher Cu signals were observed mainly from CuO (figure 3*d*–*f*).

**Figure 4.**
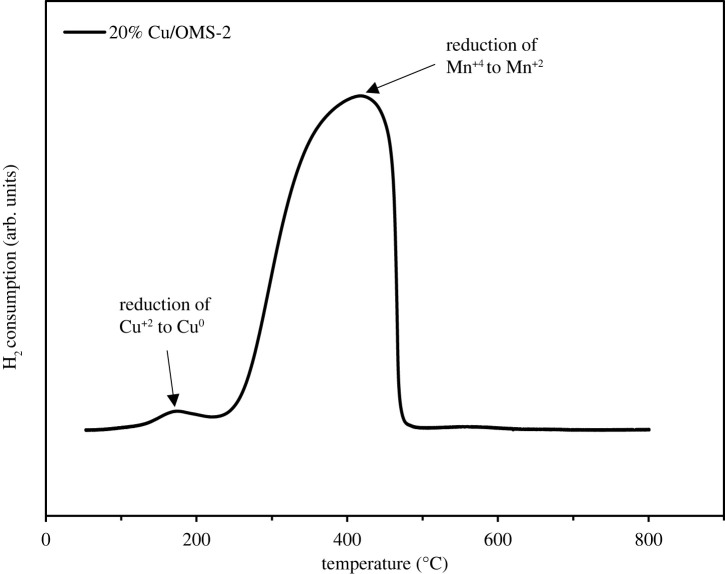
H_2_-TPR profile of 20% Cu/OMS-2.

The redox ability of the catalyst 20% Cu/OMS-2 was investigated using H_2_-TPR analysis, and the profile is presented in figure 4. The support OMS-2 represented peaks from 390 to 400°C which is attributed to the reduction of Mn^+4^ to Mn^+2^ [26]. Moreover, the peak at 190°C coincides with the reduction of CuO to Cu [27].

### 3.2. Hydrogenation of levulinic acid to γ-valerolactone

#### 3.2.1. Effect of copper loading on catalytic activity

The efficacy of OMS-2 as a catalyst and increasing Cu loading (5–20 wt%) doped on OMS-2 catalysts was compared in the hydrogenation of LA to GVL using water as a solvent.

**Figure 5.**
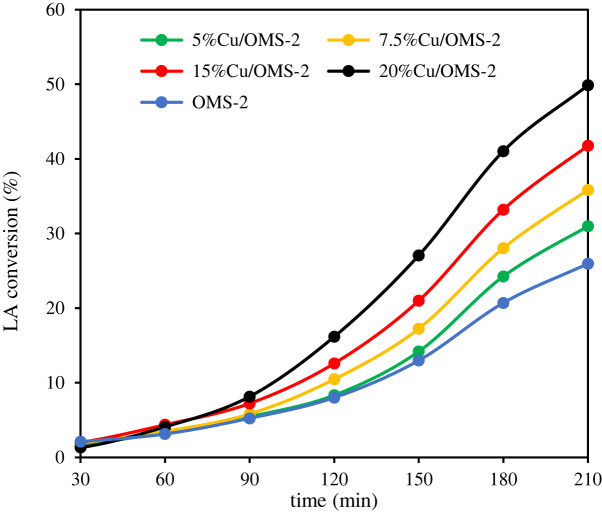
Efficacy of OMS-2 and different Cu loadings (wt%) in Cu/OMS-2 catalysts in LA hydrogenation. Reaction conditions: LA 1 g (0.009 mol), water as solvent 30 ml, catalyst 0.1 g (particle sizeless than 45 μm), temperature 160°C, H_2_ pressure 10 bar, reaction time 3.5 h.

The hydrogenation of LA to GVL proceeds in two steps: (i) dehydration of LA to α-AL, which is followed by (ii) hydrogenation of α-AL to GVL. It has been confirmed from earlier studies that OMS-2 acts as a good support with redox characteristics and structural properties to carry out the hydrogenation reaction [17]. From figure 5, the conversion of LA increased from 35 to 50% with increasing copper loading from 5 to 20 wt%. The selectivity of GVL (approx. 99%) remains unchanged with the increase in copper loading.

### 3.3. Optimization of process parameters using design of experiment

DoE methods have been influential in research and development in enhancing and improving the methodology by minimizing the number of iterations required. Response surface methodology (RSM) combines mathematical and statistical techniques to analyse and model problems to optimise one specific factor, which is governed by several other variables [28]. Central composite design (CCD) is the most popular RSM design for experimental data optimisation because of its efficiency and flexibility to provide the effects of various factors influencing the reaction with minimum iterations. CCD evaluates the co-efficient of the model equation for the reaction. The statistical software Design Expert 12.0.1.0 (Stat-Ease Inc., Minneapolis, USA) was used for the design of experiments (DoE).

The experimental runs were designed to a 2^4^ factorial design with four design independent variables, namely: hydrogen pressure in bar (X_1_), temperature in °C (X_2_), catalyst loading in wt% (X_3_) and substrate concentration in mol L^-1^ (X_4_) with low (−1) and high (+1) levels as shown in table 2. The total number of experiments (runs) was calculated by the simple formula [30 = 2*k* + 2*k* + 6], where *k* is the number of independent variables (*k* = 4). Responses selected were LA conversion and GVL yield.

**Table 2.** Actual values for the four-factor CCD.

factor	name	units	minimum	maximum	coded low	coded high	mean
A	H_2_ pressure	bar	5.00	25.00	−1–10.00	+1–20.00	15.00
B	temperature	°C	160.00	200.00	−1–170.00	+1–190.00	180.00
C	substrate concentration	mol L^-1^	0.34	1.03	−1–0.34	+1–1.03	0.68
D	catalyst loading	wt%	5.00	25.00	−1–10.00	+1–20.00	15.00

Thirty designed experiments were performed in a randomized manner for minimum errors. Table 3 demonstrates the response from the DoE optimized model plotted against the data from the experimental runs, as shown below. This equation is also applied to develop the response surface plots which present the effect of the four parameters on the GVL yield.

**Table 3.** Experimental against predicted responses. Coded model equation: GVL yield (%) = 33.26 + 15.05A + 5.16B – 5.02C + 7.84D + 8.24AB – 7.96AC + 3.79AD – 1.14BC + 1.49BD + 2.28CD −1.04A^2^ −3.54B^2^ −0.8614C^2^ – 1.50D^2^

standard	actual GVL yield (%)	predicted GVL yield (%)	residual
1	35.01	33.26	1.75
2	10.20	13.58	−3.38
3	36.86	39.86	−3.00
4	13.93	11.58	2.35
5	57.93	56.05	1.88
6	55.27	59.18	−3.91
7	33.23	33.26	−0.0267
8	2.07	−1.02	3.09
9	9.45	9.65	−0.1996
10	50.61	47.68	2.93
11	82.90	79.73	3.17
12	26.25	33.26	−7.01
13	39.77	42.94	−3.17
14	61.01	58.04	2.97
15	4.93	3.70	1.23
16	0.2800	2.18	−1.90
17	21.94	19.76	2.18
18	11.60	8.79	2.81
19	9.14	9.99	−0.8513
20	24.76	25.24	−0.4783
21	21.31	23.25	−1.94
22	19.76	17.81	1.95
23	25.80	29.44	−3.64
24	27.64	28.54	−0.9033
25	34.60	33.26	1.34
26	10.81	10.54	0.2683
27	34.35	33.26	1.09
28	36.10	33.26	2.84
29	3.83	3.14	0.6933
30	29.79	31.95	−2.16

**Figure 6.**
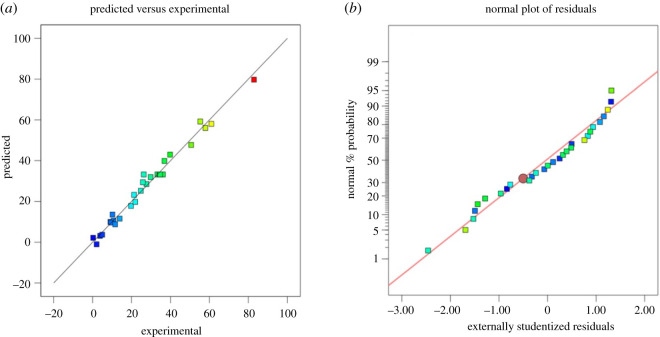
(*a*) Experimental percentage (%) yield of GVL against predicted values and (*b*) normal probability plot of residuals.

The diagnostic plots can be applied to analyse the DoE model fit. Figure 6*a* depicts the predicted and experimental results in a scatter diagram and figure 6*b* demonstrates the normal probability plot. The distribution of residuals as seen from the graph shows a straight line, the outliers are within the acceptable limits.

The yield of GVL (%) can be associated with the H_2_ pressure (A), temperature (B), substrate concentration (C) and catalyst loading (D) using analysis of variance (ANOVA) for a quadratic model. The terms A, B, C, D, AB and AC were found to be the significant model terms, with all *p*-values of less than 0.05 as shown in table 4. The H_2_ pressure was established as the most important variable (*F*-value = 406.36), followed by catalyst loading (*F*-value = 110.27), the interaction between temperature and pressure (*F*-value = 81.13), the interaction between pressure and LA to water ratio (*F*-value = 75.8) and finally temperature (*F*-value = 47.81), and LA to water ratio (*F*-value = 45.28). The *R*^2^ value evaluated from ANOVA, which indicates the correlation between the experimental values and predicted values, was calculated as 0.98 which confirms that the model is highly significant due to its low *p*-value and with high adjusted *R*^2^ value of 0.96.

**Table 4.** ANOVA plot for response surface quadratic (RSM) model.

source	sum of squares	d.f.	mean square	*F*-value	*p*-value
model	11001.43	14	785.82	58.74	<0.0001
A-pressure	5435.76	1	5435.76	406.36	<0.0001
B-temperature	639.53	1	639.53	47.81	<0.0001
C-concentration	605.71	1	605.71	45.28	<0.0001
D-catalyst loading	1475.02	1	1475.02	110.27	<0.0001
AB	1085.21	1	1085.21	81.13	<0.0001
AC	1013.94	1	1013.94	75.80	<0.0001
AD	230.36	1	230.36	17.22	0.0009
BC	20.68	1	20.68	1.55	0.2328
BD	35.55	1	35.55	2.66	0.1239
CD	83.13	1	83.13	6.21	0.0249
A^2^	29.89	1	29.89	2.23	0.1557
B^2^	343.02	1	343.02	25.64	0.0001
C^2^	20.35	1	20.35	1.52	0.2364
D^2^	61.62	1	61.62	4.61	0.0486
residual	200.65	15	13.38		
lack of fit	137.40	10	13.74	1.09	0.4940
pure error	63.25	5	12.65		
cor total	11202.09	29			

**Figure 7.**
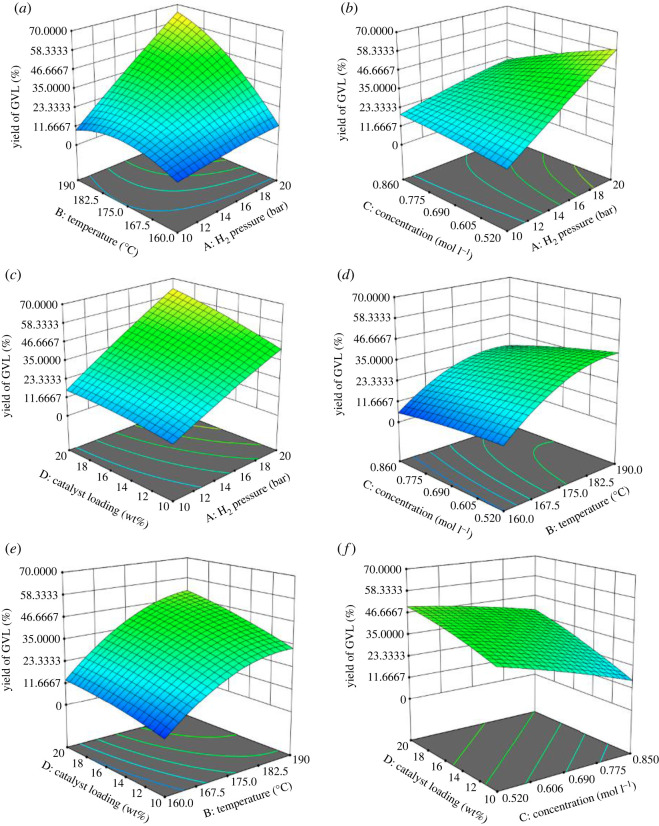
(*a*) Three-dimensional response plot for the effect of temperature and H_2_ pressure on GVL yield; substrate concentration 0.59 mol L^−1^, catalyst loading 15.1 wt%; (*b*) three-dimensional response plot for the effect of substrate concentration and H_2_ pressure on GVL yield; 180°C temperature, 15.1 wt% catalyst loading; (*c*) three-dimensional response plot for the effect of catalyst loading and H_2_ pressure; 180°C temperature, substrate concentration 0.59 mol L^−1^; (*d*) three-dimensional response plot for the effect of temperature and substrate concentration; 15 bar of H_2_ pressure, catalyst loading 15.1 wt%; (*e*) three-dimensional response plot for the effect of catalyst loading and temperature; 16.1 bar of H_2_ pressure, substrate concentration 0.6 mol L^−1^; (*f*) three-dimensional response plot for the effect of substrate concentration and catalyst loading; H_2_ pressure 17 bar, temperature 178.4°C.

Figure 7*a* depicts the effect of temperature and pressure on the percentage yield of GVL, using substrate concentration of 0.59 mol L^−1^ and catalyst loading of 15.1 wt%. As H_2_ pressure increases from 10 to 20 bar, the percentage yield of GVL increases; however, it is not as distinct with the increase of temperature from 170 to 190°C. An increase of H_2_ pressure at high temperature can be seen to result in a significant increase in the GVL yield to over 58%. The interaction of these two factors has a marked effect on GVL yield. Figure 7*b* shows the effect of substrate concentration and H_2_ pressure on the GVL yield at 180°C temperature and 15.1 wt% catalyst loading. It can be observed that with the increase of pressure, the GVL yield increases, while substrate concentration had little effect on the yield of GVL. Figure 7*c* shows the effect of catalyst loading and H_2_ pressure at a temperature of 180°C and substrate concentration of 0.59 mol L^−1^. An increase in pressure and catalyst loading resulted in high GVL yield. Figure 7*d* shows the effect of temperature and substrate concentration at 15 bar of H_2_ pressure and catalyst loading of 15.1 wt%. It can be seen that with the increase in temperature, the yield of GVL increased; while the increase in substrate concentration resulted in the decrease of GVL yield. Figure 7*e* shows the effects of catalyst loading and temperature at 16.1 bar of H_2_ pressure and substrate concentration of 0.60 mol L^−1^. As noticed before, with an increase in temperature there is an increase in GVL yield, and the same trend is seen with the increase in catalyst loading. Figure 7*f* shows the effect of substrate concentration and catalyst loading with H_2_ pressure of 17 bar and temperature of 178.4°C. It can be seen that with an increase in substrate concentration the yield of GVL decreases, while an increase in the catalyst loading resulted in an increase of GVL yield.

**Figure 8.**
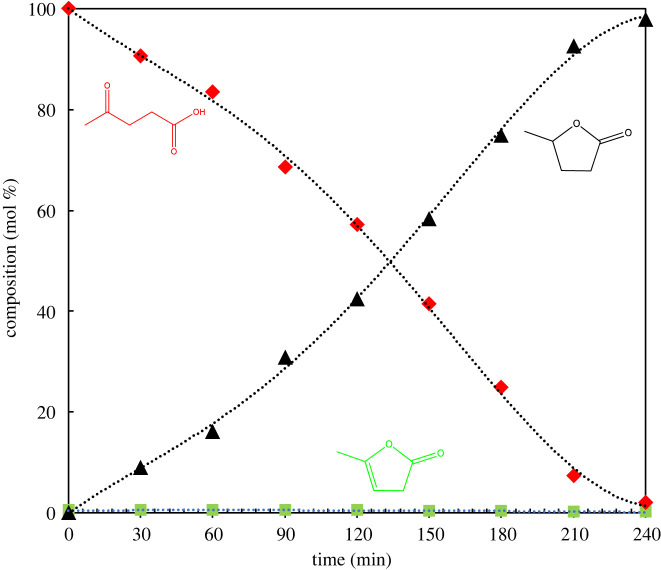
A typical reaction composition-time profile of LA hydrogenation using optimized process conditions by DoE approach. Reaction conditions: LA 1.8 g (0.016 mol), water as solvent 30 ml, 20 %Cu/OMS-2 catalyst 0.36 g (particle sizeless than 45 μm), temperature 190°C, H_2_ pressure 20 bar, reaction time 4 h.

Using the above DoE approach, the process parameters for LA hydrogenation were optimized and a control reaction was performed using the optimized conditions. Figure 8 shows the typical reaction composition-time profile for LA hydrogenation under DoE optimized conditions, using 20 %Cu/OMS-2 as a catalyst at 190°C temperature and 20 bar hydrogen pressure. The reaction was completed within 4 h with complete conversion of LA and 98% selectivity to GVL.

### 3.4. Catalyst reusability

**Figure 9.**
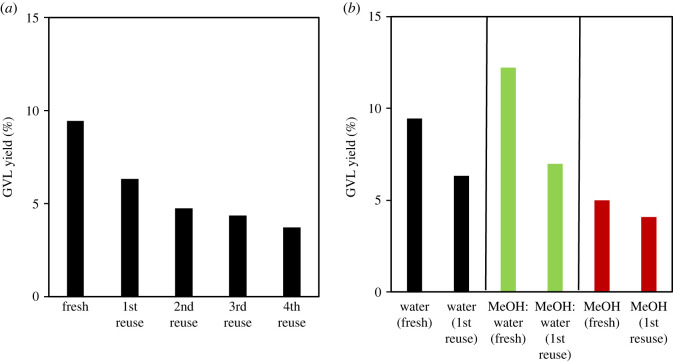
Effect of catalyst reusability on the hydrogenation of LA, (*a*) %Yield of GVL in water as solvent, up to five cycles, and (*b*) %Yield of GVL in individual water, 10 mol% methanol in water and methanol as solvents.

The reusability of 20 %Cu/OMS-2 catalyst was studied over five consecutive cycles in LA hydrogenation using water as the solvent. The catalyst reusability tests were performed under low conversions of LA to reflect upon the true catalyst stability, instead of using higher catalyst loadings to achieve higher LA conversions. The activity of the catalyst decreased noticeably after the first cycle, and thereafter the degree of activity loss diminished over subsequent recycles, as shown in figure 9*a*. This trend in the loss of catalyst activity may be attributed to the loss of loosely bounded CuO from the catalyst surface during the first cycle and the loss of catalyst due to attrition during subsequent recycles. ICP analysis of the reaction mixture did not show Cu in solution, but Cu depositions on the reactor stirrer were noticeable. It's likely that the leached CuO species were reduced to Cu and deposited on the stirrer. In previous studies, Hengne *et al.* [14] also observed copper leaching from ZrO_2_ and Al_2_O_3_ as supports while carrying out LA hydrogenation. Copper leaching while using water as a solvent was attributed to the formation of a soluble metal carboxylate complex on reaction with LA. Ishikawa *et al*. [15] also observed a steady decline of catalytic activity over four recycles with Cu species leaching from ZrO_2_ catalysts in LA hydrogenation. In control experiments, we also evaluated 10 mol% methanol in water mixture and individual methanol as solvents for LA hydrogenation. As shown in figure 9*b*, in methanol-water solvent mixture higher yields of GVL were obtained, in comparison with water and methanol as individual solvents. In methanol-water and methanol as solvents, the formation of methyl levulinate as an intermediate helps to decrease the leaching of Cu. The use of 10 mol% methanol-water mixture as a solvent showed improved GVL yield. The observed reaction rates were relatively higher in 10 mol% methanol-water mixture as solvent, in comparison to both individual methanol and water as solvents. These results are consistent with our previous studies in liquid-phase hydrogenations in alcohol-water mixtures, wherein we demonstrate that the catalytic performance can be tailored through controlling the structural dynamics of the solvent. Tuning the solvent mixtures helps to lower the activation energy barriers as well as increase the proton diffusion coefficient [21,22].

**Figure 10.**
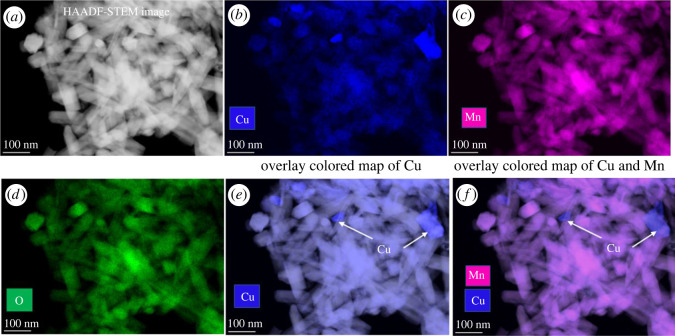
20 wt% Cu/OMS-2 used catalyst (*a*) HAADF-STEM image, and (*b–d*) corresponding EDX elemental maps of Cu, Mn and O, and (*e,f*) an overlay false colour image of Cu and Cu-Mn on the HAADF image.

**Figure 11.**
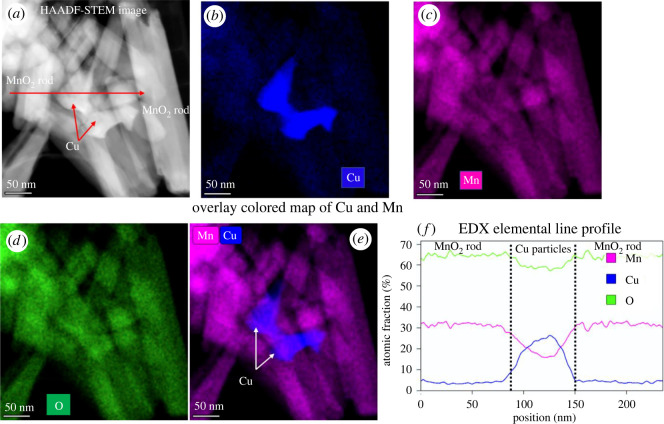
20 wt% Cu/OMS-2 used catalyst (*a*) high-magnification HAADF-STEM image, and (*b–d*) corresponding EDX elemental maps of Cu, Mn and O, and (*e*) an overlay image of Cu and Mn. (*f*) Line profile (atomic percentage) of elements across the MnO_2_ and Cu particles area (follow the marked red arrow in the HAADF-STEM image).

In a separate control experiment, we recovered the used catalyst after the reaction and compared copper distribution of fresh and spent catalyst. A reaction with optimized conditions (T = 190°C, *p* = 20 bar H_2_, LA = 1.8 g (0.015 mol), catalyst loading (20% Cu/OMS-2) = 0.36 g, water = 30 ml, time = 1 h) was performed and the catalyst was recovered using centrifugation of the reaction mass. After overnight drying at 110°C, the sample was analysed using STEM-EDX for Cu, Mn and O (figure 10). We do see slightly more uniformity in the Cu distribution in the spent catalyst, while Mn and O are distributed homogeneously across the regions (follow the Mn and O maps in figure 10*c*,*d*). Cu can be seen as agglomerates and small particles, while the distribution seems to be improved in the spent catalyst. High-magnification elemental mapping of MnO_2_ rods and Cu particles are shown in figure 11. Both Mn and O atomic fractions look more stable across the whole area of the rods (figure 11*f*). Elemental line profile (in atomic per cent, follow the red arrow in figure 11*f*) was taken across the MnO_2_ rod-Cu particle-MnO_2_ rod. It is evident from the line profile that higher (up to 25 at%) Cu atomic fractions correlated well with the presence of Cu particle in the image. These measurements were conducted on a nickel (Ni) TEM grid to avoid any artefacts, in particular Cu from the TEM grids.

## 4. Conclusion

Manganese oxide-based OMS-2 and Cu-doped OMS-2 were found to be active and selective catalysts for the efficient hydrogenation of LA to GVL using water as a solvent. Among the various Cu loadings on OMS-2 studied, 20 wt%Cu/OMS-2 was found to be the most active catalyst. Process parameters for selective hydrogenation of LA were optimized using the DoE approach with RSM. Among the various reaction parameters used for optimization, H_2_ pressure showed the most significant influence followed by catalyst loading on selectivity to GVL. The model also showed interaction between temperature and H_2_ pressure. Using the optimized process parameters, complete conversion of LA could be achieved with greater than 99% selectivity to GVL. The 20 wt%Cu/OMS-2 catalyst could be easily recovered and re-used over five cycles. 10 mol% methanol in water as solvent showed higher reaction rates for LA hydrogenation than individual methanol and water as solvent.

## Data Availability

Required experimental data and catalyst characterizations are already included in the manuscript.
